# The management and clinical knowledge of headache disorders among general practitioners in Norway: a questionnaire survey

**DOI:** 10.1186/s10194-021-01350-3

**Published:** 2021-11-11

**Authors:** Espen Saxhaug Kristoffersen, Kashif Waqar Faiz, Jakob Møller Hansen, Erling Andreas Tronvik, Jan C. Frich, Christofer Lundqvist, Bendik Slagsvold Winsvold

**Affiliations:** 1 Department of General Practice, University of Oslo, PO Box 1130, Blindern, 0318 Oslo, Norway; 2 Department of Neurology, Akershus University Hospital, Lørenskog, Norway; 3 Department of Research and Innovation, Division of Clinical Neuroscience, Oslo University Hospital, Oslo, Norway; 4 Danish Knowledge Centre on Headache Disorders, Rigshospitalet-Glostrup, University, of Copenhagen, Glostrup, Denmark; 5 Department of Neuromedicine and Movement Science, NTNU Norwegian University of Science and Technology, Trondheim, Norway; 6 Department of Neurology, National Advisory Unit on Headaches, St. Olavs Hospital, Trondheim, Norway; 7 Department of Health Management and Health Economics, Institute of Health and Society, University of Oslo, Oslo, Norway; 8 Health Services Research Unit, Akershus University Hospital, Lørenskog, Norway; 9 Institute of Clinical Medicine, University of Oslo, Oslo, Norway; 10 Department of Neurology, Oslo University Hospital, Oslo, Norway

**Keywords:** Migraine, Medication-overuse headache, Education, Quality, Guidelines, Primary care

## Abstract

**Background:**

General practitioners (GPs) diagnose and manage a majority of headache patients seeking health care. With the aim to understand the potential for clinical improvement and educational needs, we performed a study to investigate Norwegian GPs knowledge about headache and its clinical management.

**Methods:**

We invited GPs from a random sample of 130 Norwegian continuous medical education (CME) groups to respond to an anonymous questionnaire survey.

**Results:**

367 GPs responded to the survey (73% of invited CME groups, 7.6% of all GPs in Norway). Mean age was 46 (SD 11) years, with an average of 18 (SD 10) years of clinical experience. In general the national treatment recommendations were followed, while the International Classification of Headache Disorders and other international guidelines were rarely used. Overall, 80% (*n* = 292) of the GPs suggested adequate prophylactic medication for frequent episodic migraine, while 28% (*n* = 101) suggested adequate prophylactic medication for chronic tension-type headache (CTTH). Half (52%, *n* = 191)) of the respondents were aware that different types of acute headache medication can lead to medication-overuse headache (MOH), and 59% (*n* = 217) knew that prophylactic headache medication does not lead to MOH. GPs often used MRI in the diagnostic work-up. GPs reported that lack of good treatment options was a main barrier to more optimized treatment of headache patients.

**Conclusion:**

The knowledge of management of CTTH and MOH was moderate compared to migraine among Norwegian GPs.

**Supplementary Information:**

The online version contains supplementary material available at 10.1186/s10194-021-01350-3.

## Background

The focus on headache in the curriculum at medical schools and in specialist training throughout the world is limited [1–3]. Insufficient education, training and knowledge about headache may be a cause of diagnostic failure, inappropriate treatment, and low patient satisfaction among headache patients [4]. Headache is one of the main reasons why patients contact a general practitioner (GP), and because most people with headaches are well-managed by the GPs, only a small proportion of patients are referred to more specialized care [5–8]. GPs play a key role both in treatment and as gatekeepers for referrals to specialist care [9]. However, for this to function, it is imperative that the GP has sufficient knowledge and validated tools for everyday use. The burden of headache and migraine is high and the findings suggest that diagnosis and management in the health care system is still limited [10–12]. International guidelines and National recommendations for diagnosis and management developed by the National Advisory Unit on Headache in Norway exist [12–15], but data is very limited on how GPs actually experience and manage headache patients [16, 17]. In the present exploratory descriptive study we investigate the knowledge among Norwegian GPs about headache and the clinical management of patients with headache.

## Methods

### Design and setting

Primary health care in Norway are provided by GPs in a patient-list system. More than 99.8% of people living in Norway (5.4 million) use the GP patient-list system [18]. The average patient list consists of approximately 1100 patients per GP. Norwegian GPs are on average 47.2 years old, 45.8% are women and 63.2% are certified specialists in general practice [18].

In 2017 it became mandatory for all GPs in Norway to be a certified GP specialist or under training to become a certified GP specialist. At the time of this survey the training program to become a certified GP specialist includes 1.5 years of internship (usually internal medicine, general surgery, and general practice), followed by 4 years of GP training, of which 1 year must be spent in another clinical specialty. In addition, clinical training courses and individual supervision must be completed; however, a headache course is optional. GP specialists must also participate in mandatory peer continuous medical education (CME) groups to be re-certified every fifth year. In everyday clinical practice the responsibilities and working situation does not differ between GPs under training and those certified as GP specialists.

We invited GPs to respond to an anonymous questionnaire survey between 2018 and 2019 through invitations to a sample of 130 Norwegian CME groups. The CME groups were invited based on a representative geographical distribution with both rural and urban GPs. The CME groups were invited by email and received one reminder. The administrator (one of the GPs) of each CME group distributed the invitation to the others. CME groups usually consists of 3–5 GPs, but there exists no updated list for all GPs in CME groups in Norway. Participants were required to complete questionnaires individually in a web-based questionnaire.

### Questionnaire

We developed a questionnaire (supplement 1) based on available literature and the authors’ experience in health service research, general practice and headache management [3, 17]. The questionnaire covered background variables and the participants responded to a short multiple-choice and open questions about knowledge of and experience in the management of headache, use of the national treatment recommendations and the International Classification of Headache Disorders 3rd edition (ICHD-3) and barriers to optimised treatment.

We used a 3-item scale with the options “good”, “moderate” and “poor” to assess self-rated knowledge of migraine, tension-type headache, medication-overuse headache (MOH), and cluster headache/trigeminal autonomic cephalalgias (TACs).

We asked GPs how often they used headache diaries, imaging, web-sites (BMJ Best Practice and UpToDate are both available for free for clinicians in Norway), national treatment recommendations, and the ICHD-3, with the response options i) *every time*, ii) *two out of three times*, iii) *half the time*, iv) *one out of three times/rarely*, and v) *never*. The categories were re-classified into “> 2/3”, “half the time”, and “< 1/3”, due to few responses in the categories *every time* and *never*.

Two different headache cases were used to assess the participants’ suggestions for medical treatment of patients with i) chronic tension-type headache (CTTH), and ii) frequent episodic migraine (1–2 migraine attacks every week), two of the most seen headache types in general practice. Several questions concerned MOH.

To assess the main barriers to optimised management of patients with headache and reasons for referral, we asked the participants to rank pre-specified statements.

### Outcomes

Outcomes were mainly categorised descriptive data based on the described variables. In addition, demographic, practice-related and medical training-related predictors for pre-defined logistic answers (yes/no) regarding knowledge of acute and prophylactic medical treatment of CTTH, migraine and MOH for the following outcomes were analysed:

Knowledge of CTTH prophylaxis was defined as yes if the participant suggested a tri-cyclic antidepressant as the prophylactic medication of choice in CTTH.

Knowledge of migraine prophylaxis was defined as yes if the participant suggested a beta-blocker, lisinopril, candesartan, topiramate, amitriptyline, valproate or Botulinum Toxin A as the prophylactic medication of choice in frequent episodic migraine.

Knowledge of prophylactic medications and MOH was defined as yes if the participant answered correctly that commonly used prophylactics (antiepileptic drugs, anti-hypertensive drugs, antidepressant drugs, and botulinum toxin A) do not induce MOH.

Knowledge of acute medications and MOH was defined as yes if the participant answered correct that simple analgesics, combination analgesics, opioids and triptans may induce MOH. As ergotamine is very rarely used in Norway, this medication group was not included.

### Statistical analyses

For descriptive data, proportions, means, and standard deviations (SD), or 95% confidence intervals (CI) are given. Groups were compared using the *t*-test (continuous data) or the *χ*^2^ test (categorical data).

We used multiple logistic regression analysis to evaluate the effect of age, gender, certified specialist in general practice, number of patients on the GP list, and whether the GP had attended a headache course on the outcomes (yes/no) pertaining to knowledge of adequate prophylactic medication use for CTTH and frequent episodic migraine as well as prophylactic and acute medication use in MOH.

The results of the questions of barriers to optimal management and referrals are presented descriptively as proportions, medians and interquartile range (IQR). Comparisons between the ranked statements were done by Wilcoxon Matched-Pairs Signed Ranks test.

Statistical significance was defined by *p* < 0.05, using a two-sided test. As this was an exploratory descriptive study, we did not perform an a priori power calculation or adjust for multiple comparisons. Statistical analyses were performed using IBM SPSS Statistics, Version 27.00 (SPSS Inc., Chicago, IL, USA).

## Results

In total, 367 GPs responded to the survey. Respondents were recruited from 95 (73%) out of 130 invited CME groups. Among the CME groups that did not participate, 26 (20%) did not respond to the invitation, and nine (7%) declined to take part in the study. The mean age of respondents was 46 (range 25–70) years, half (49%) of the respondents were women, and 71% were certified specialists. On average, the respondents had almost 15 years of experience in general practice. Table 1 presents descriptive data about the respondents.

**Table 1:** Descriptive data of the participants (*N* = 367)

Sex n (%)	
Women	179 (49)
Men	188 (51)
Age, mean (SD)	46.0 (10.5)
Years as physician, mean (SD)	17.8 (10.4)
Years as general practitioner, mean (SD)	14.4 (10.3)
Certified specialist in general practice, n (%)	
Yes	262 (71)
No	105 (29)
Years as certified specialist in general practice, mean (SD)	12.3 (9.2)
Number of patients on list, mean (min-max)	1133 (400–2200)
In-person consultations per day, mean (SD)	18.7 (3.8)
Attended headache course, n (%)	
Yes	83 (23)
No	284 (77)

### Knowledge

**Fig. 1:**
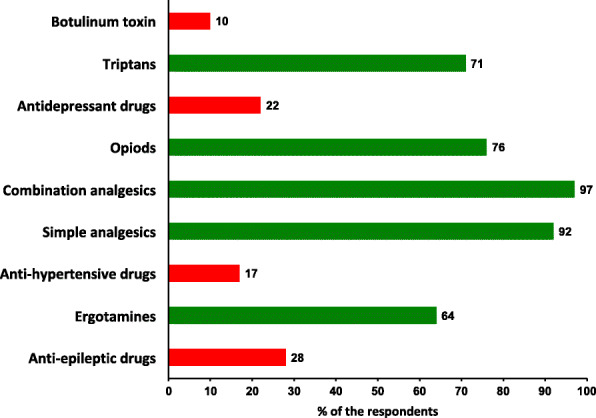
Percentage of the participants who stated that each of the given medication can lead to medication-overuse headache. Green bars represent those medications that are known to cause medication-overuse headache, while red bars represent those that do not

Knowledge of which medications may lead to MOH is reported in Fig. 1. Fifty-nine percent (*n* = 217) answered all questions on MOH (i.e. acute medications and prophylaxis) correctly, but many wrongly stated that one of the most commonly used headache prophylactics could lead to MOH (17% (*n* = 63) for anti-hypertensives, 28% (*n* = 101) for anti-epileptics, and 22% (*n* = 79) for antidepressant drugs). Fifty-nine% (n = 217) knew that none of the prophylactic headache medication induce MOH. More than 90% (*n* = 332) of the respondents knew that simple analgesics and combination analgesics may lead to MOH. Overall, 52% (*n* = 191) of the respondents answered all questions on acute headache medication correct i.e. simple analgesics, combination analgesics, opioids and triptans may induce MOH. Furthermore, 24% (*n* = 87) wrongly stated that highly potent opioids, and 29% (*n* = 106) that triptans, cannot lead to MOH. A significantly larger proportion of GP specialists compared to non-specialists answered all questions about medication and MOH correctly (64% (*n* = 167) vs. 48% (*n* = 50), *p* = 0.005).

**Fig. 2:**
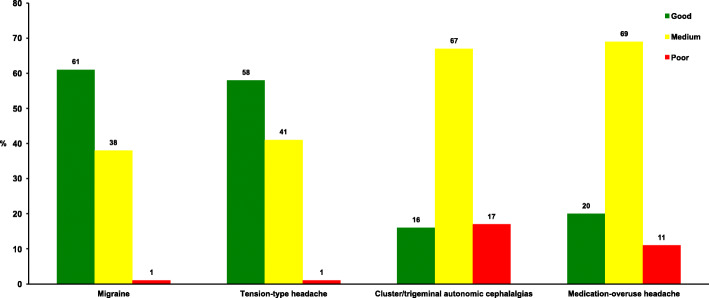
Percentage of the participants responding good (green), medium (yellow), or poor (red) to the question “How do you rate your own knowledge of migraine, tension-type headache, cluster headache/trigeminal autonomic cephalalgias, and medication-overuse headache?”

Figure 2 shows that self-reported knowledge was reported as good concerning migraine and tension-type headache in two out of three GPs, whereas few GPs stated that they had good general knowledge about cluster headache, TACs and MOH. There were no gender differences in self-reported knowledge, but specialists reported better knowledge about migraine, cluster headache, TACs and MOH than non-specialists (*p* < 0.01).

A higher proportion of those with good self-reported knowledge of migraine answered the questions about prophylactic treatment of migraine correct compared to those with poor self-reported knowledge (80% vs 33%, *p* = 0.043). There was a non-significant difference in the self-reported knowledge of TTH and the reported correct use of preventives in CTTH. Correct use was reported by 32% (good self-reported knowledge), 22% (medium self-reported knowledge) and 0% (poor self-reported knowledge), *p* = 0.06 for TTH. There was a significant difference in the self-reported knowledge of MOH and the actual knowledge about acute medication overuse and use of preventives in MOH. Adequate knowledge of MOH was found in 72% (good self-reported knowledge), 57% (medium self-reported knowledge) and 51% (poor self-reported knowledge), *p* = 0.033. Altogether, the proportion that self-reported good knowledge about migraine, TTH and MOH and answered the questions about these disorders satisfactorily were 80%, 32% and 72%, respectively.

In the logistic regression models, none of the tested variables were associated with a higher proportion of respondents with knowledge about prophylactic headache medication (CTTH, migraine and MOH). The only significant finding was that GP specialists had an increased odds of 1.9 (95% CI 1.1; 3.3, *p* = 0.02) for knowing that acute headache medication may induce MOH.

### Clinical diagnostics and management

The most frequent response to the question “Do you find headache to be a clinically difficult professional challenge (1=difficult and 4=easy)” was alternative 2 (50%), followed by alternative 3 (41%). A majority of 59% (*n* = 215) used the national treatment recommendations for headache in > 2/3 of consultations. Other international resources such as ICHD-3, BMJ Best Practice, UpToDate or Google search were used rarely (Table 2). There were no differences between specialists and non-specialists.

**Table 2:** General Practitioners’ use of diagnostic and treatment tools for diagnosis and follow-up of headache patients. All figures are numbers (%)

	All (N = 367)
Headache diary for diagnosis	
> 2/3 times	127 (35)
1/2 times	93 (25)
< 1/3 times	147 (40)
Headache diary for follow-up	
> 2/3 times	111 (30)
1/2 times	77 (21)
< 1/3 times	179 (49)
National treatment recommendations	
> 2/3 times	215 (59)
1/2 times	65 (18)
< 1/3 times	87 (24)
International Classification of Headache Disorders	
> 2/3 times	30 (8)
1/2 times	11 (3)
< 1/3 times	326 (89)
BMJ Best Practice and/or UpToDate	
> 2/3 times	9 (3)
1/2 times	11 (3)
< 1/3 times	347 (95)
Google search	
> 2/3 times	7 (2)
1/2 times	15 (4)
< 1/3 times	345 (94)

Headache diaries were used regularly (i.e. in > 2/3 of consultations) by 35% (*n* = 127) for diagnostic purposes and by 30% (*n* = 110) for follow-up. Thirty-four percent reported that they received the diary back from the patients in 2/3 of follow-ups. There were no significant differences in the use of diaries between specialists and non-specialists.

Thirty-three percent (*n* = 123) of participants responded that they asked patients about disability, social functioning, and sick leave in every consultation, and an additional 27% (*n* = 99) asked about these factors in > 2/3 consultations. Seventeen percent (*n* = 63) asked these questions in 1/3 of consultations or fewer.

Table 3 shows participants’ reported use of imaging. Almost all GPs used imaging for headaches with focal neurological symptoms, and 84% used imaging if the headache was not responding to treatment. Sixty-two percent of participants reported using imaging if the patient had concerns and anxiety about brain tumor or other intracranial pathology. Overall, GPs rated MRI as more useful than CT, 59% found MRI useful in headache diagnostics in at least half of the patients, while the corresponding number for CT was 21%.

**Table 3:** The use of imaging of headache patients among general practitioners. All figures are numbers (%)

	All (N = 367)
*Do you usually use CT/MRI for the following?*	
All new-onset headache (not acute)	
Yes	23 (6)
No	344 (94)
Headache not responding to treatment	
Yes	308 (84)
No	59 (16)
Headache with focal neurological symptoms	
Yes	362 (99)
No	5 (1)
Worsening of a pre-existent headache	
Yes	236 (64)
No	131 (36)
If patients have concerns and anxiety about brain tumor or other intracranial pathology	
Yes	228 (62)
No	139 (38)
Neck pain with concomitant headache	
Yes	52 (14)
No	315 (86)
*How often do you?*	
Use CT for new-onset headache (not acute headache)	
> 2/3 times	7 (2)
1/2 times	19 (5)
< 1/3 times	341 (93)
Use MRI for new-onset headache (not acute headache)	
> 2/3 times	66 (18)
1/2 times	56 (15)
< 1/3 times	245 (67)
Use CT for long-lasting headaches	
> 2/3 times	17 (5)
1/2 times	24 (7)
< 1/3 times	326 (89)
Use MRI for long-lasting headaches	
> 2/3 times	103 (28)
1/2 times	69 (19)
< 1/3 times	195 (53)
Find CT useful in headache investigations	
> 2/3 times	41 (11)
1/2 times	36 (10)
< 1/3 times	290 (79)
Find MRI useful in headache investigations	
> 2/3 times	153 (42)
1/2 times	64 (17)
< 1/3 times	150 (41)
Use CT or MRI to alleviate a patient’s concerns/anxiety about tumor cerebri or other intracranial pathology	
> 2/3 times	72 (20)
1/2 times	75 (20)
< 1/3 times	220 (60)

**Fig. 3:**
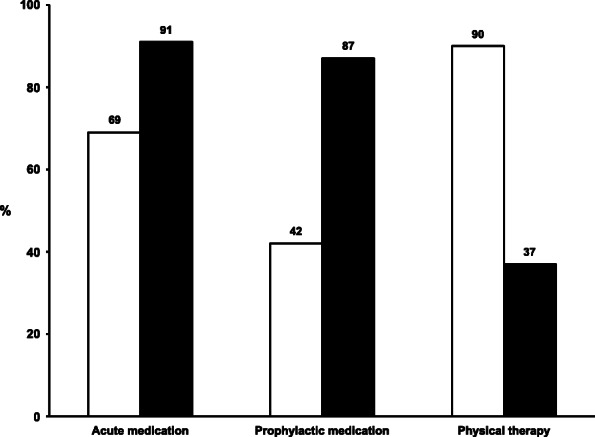
Percentage of respondents who would use different types of treatments for patients with chronic tension-type headache (white bars) and frequent episodic migraine (1–2 attacks per week) (black bars)

Figure 3 show the proportion of respondents who would treat patients with CTTH and frequent episodic migraine (1–2 migraine attacks every week) with acute headache medication prophylactic headache medication, and physiotherapy, respectively. Sixty-nine percent of respondents would treat a patient with CTTH with acute medication, with significantly fewer GP specialists than non-specialists (65% vs. 80%, *p* = 0.004). Simple analgesics (paracetamol or a combination of paracetamol and ibuprofen/other NSAIDs) were the most commonly suggested acute medications for CTTH. Among the 42% (*n* = 154) that suggested prophylactic medication for CTTH, amitriptyline was suggested by 65% (*n* = 100), but as many as 17% (*n* = 26) suggested acute headache medications also for prophylactic use (paracetamol/ibuprofen/other NSAIDs). Overall, 28% (*n* = 101) of the GPs suggested adequate prophylactic medication according to national recommendations for CTTH.

Triptans (87%, *n* = 319), ibuprofen/NSAIDs/acetylsalicylic acid (19%, *n* = 67), and paracetamol (8%, *n* = 30) were suggested for the acute treatment of frequent migraine. Prophylactic medication was suggested for frequent migraine by 87% (*n* = 318), with no differences between GP specialists and non-specialists. Candesartan (60%, *n* = 192) and beta-blockers (56%, *n* = 179) were the most commonly suggested prophylactic medications, while only 4% (*n* = 13) suggested topiramate. Overall, 80% (*n* = 292) of the GPs suggested adequate prophylactic medication according to guidelines for frequent episodic migraine.

Seventy percent (*n* = 254) of the respondents reported that they believed that non-prescription drugs were the most commonly used medication by their chronic headache patients to treat headache. Only 4% (*n* = 14) believed that their chronic headache patients used pain killers with addictive potential. The majority of GPs (54%, *n* = 185) thought that a minority (< 40%) of their chronic headache patients had medication overuse, while 32% (*n* = 117) of the GPs estimated this to be the case for 40–60% of their chronic headache patients.

Sixty percent (*n* = 219) reported MOH to be a clinical challenge among their headache patients with significantly more GP specialists than non-specialists finding MOH a clinical challenge (64% vs. 50%, *p* = 0.009). Ninety-seven percent (*n* = 356) used withdrawal as part of their treatment of MOH. Fifty-five percent (*n* = 200) recommended initial withdrawal only, whereas 43% (*n* = 156) used withdrawal combined with initial prophylactic medication. Fifty percent (*n* = 182) proposed sick-leave during the withdrawal phase and 17% (*n* = 62) recommended rescue medication as a part of the withdrawal strategy. Furthermore, 15% (*n* = 55) recommended in-patient withdrawal for patients with MOH (specialists vs. non-specialists 19% vs. 6%, *p* = 0.002).

**Fig. 4:**
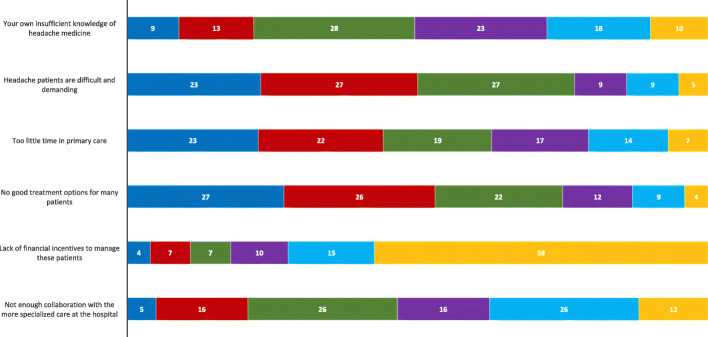
GPs’ ranking of the main barriers to optimised treatment and management of headache patients, scored on a scale of 1–6. The most important barrier is ranked from right to left as 1 (blue) and the least important as 6 (orange). The corresponding colours to score 2, 3, 4 and 5 are red, green, purple and turquoise, respectively. Numbers denote %

The GPs answered the question “What do you believe are the main barriers to optimised treatment and management of your headache patients?” (1–6, where 1 is the most important and 6 is the least important barrier) (Fig. 4). Twenty-seven percent (*n* = 96) scored “No good treatment options for many patients” as the main barrier to more optimized treatment of headache patients (median 2, IQR 1–3, *p* < 0.001 compared to all other barriers). The next two most reported barriers were “Headache patients are difficult and demanding” (median 3, IQR 2–3) and “Too little time in general practice” (median 3 IQR 2–4). Only 9% (*n* = 33) regarded their own insufficient knowledge to be the most important barrier. Lack of financial incentives to treat headache patients was perceived as the least important barrier. Gender and specialist status did not influence the ranking of barriers.

The most common reason for referring headache patients was treatment failure/lack of good treatment options in primary care (median 2, IQR 1–2, *p* < 0.001 compared to all other reasons). Diagnostic uncertainty (median 2, IQR 1–3) was the second most common reason, followed by suspicion of serious underlying cause (median 2, IQR 1–4) and the patient’s own wishes/expectations (median 3, IQR 2–4).

## Discussion

A main finding in this nationwide questionnaire-based study among GPs was large differences in the knowledge of management of CTTH and MOH compared to migraine. These findings and potential gaps are important and should be further explored as headache disorders are one of the main reasons for contact with GP, and the large majority of headache patients are treated in primary care.

### Strengths and limitations

The study sample consisted of 367 GPs recruited from a sample of 130 CME groups, and represents as many as 7.6% of all GPs in Norway (*N* = 4787) when the survey was conducted. Participants were asked to complete questionnaires individually in a web-based questionnaire; thus, cluster-effect based on CME groups is less likely. Together with a high response rate of CME groups (73%), this should ensure reasonable representativeness and generalizability. However, potential selection bias from those willing to participate will always be a limitation in such studies. The study assesses GPs´ views on headache treatment and assessment, which may differ from their actual practice. The study assesses the most common headache disorders (TTH, migraine, MOH and TACs), but the findings of knowledge and management may not be extrapolated to all other headache disorders. The questionnaire has not been validated, but is based on two similar studies conducted in the US and in Norway [3, 17].

### Interpretation of results and comparison with other studies

More than 50% of the GPs reported headache management to be clinically difficult, which underlines the importance of educating GPs to be comfortable in diagnosing and treating headache disorders [16, 19]. No diagnostic tests exist for headache disorders, and diagnoses are primarily based on good history taking and clinical examination. All treatment guidelines are based on specific diagnoses, thus it is of uttermost importance to make the correct diagnosis. Although previous studies among GPs have shown that many patients do not receive a specific headache diagnosis, our finding that only 8% used the diagnostic headache criteria (ICHD-3) on a regular basis was lower than expected [17, 20–23]. Still, this finding is in line with previous studies that have found that many physicians do not use formal diagnostic classifications as they find them impractical for use in daily practice [21, 24]. Headache diaries, which are recommended for diagnosis and follow-up, were used regularly by approximately one in three GPs.

Neuroimaging is not an essential part of headache investigations and should be reserved for those with red flags indicating secondary headaches [12, 25]. Several studies have shown that neuroimaging is routinely ordered despite a typical headache history and normal clinical examination [26, 27]. Almost all the GPs in our study used imaging for headache with focal neurological symptoms, a typical “red flag”, which is in line with the guidelines. Also, few GPs used imaging in patients with concomitant neck pain and headache, where imaging is typically of little value. This contrasts with findings from a large multi-national study [28]. Almost two in three GPs used imaging if the patients had concerns and anxiety about brain tumor or other intracranial pathology, suggesting that patient pressure and expectations play a role in decision of whether or not to do neuroimaging. This may be a contributing explanation for the high use of neuroimaging in headache patients. Unnecessary imaging comes with a cost. In addition to the direct costs of the procedure, it may lead to unnecessary fear, repeated investigations, and follow-up of incidental findings. Despite its common use, it is noteworthy that the GPs did not necessarily find imaging very useful.

Effective treatments exist for the most common primary headache disorders, migraine and tension-type headache [12, 29]. About one in three GPs reported that they had good knowledge about migraine and TTH, and > 96% reported good or medium knowledge about migraine and TTH. The reported treatment suggestions for migraine were largely in line with this self-reported good knowledge of migraine. The vast majority suggested triptans for acute treatment of migraine and adequate prophylactic medication was suggested for frequent episodic migraine by as many as 87%. Candesartan and beta-blockers are two of the first line choices in most treatment guidelines and were the most commonly suggested prophylactic medications [12–14, 30]. Few GPs suggested topiramate and Botulinum toxin type A. Topiramate has class I evidence and should probably be used more by patients with migraine. The use of Botulinum toxin type A is highly restricted in Norway, and in the study period they could only be prescribed by neurologists to selected chronic migraine patients who had failed > 3 prophylactic medications. The present study was conducted prior to the introduction of the CGRP-antibodies in Norway, however, these are now subject to similar limitations and prescription must go through specialists in neurology.

As opposed to the case for migraine treatment, less than half of the respondents suggested prophylactic medication for CTTH, and only 28% suggested what would be regarded as the first choice in the treatment of CTTH (amitriptyline). Even more worrying, one-third of the participants wrongly stated that the most commonly used headache prophylactics (anti-hypertensives, anti-epileptics, and antidepressant drugs) could lead to MOH. This misinterpretation can lead to a worse clinical outcome, and may be one of several explanations for under-use of prophylactics in headache disorders [5, 12, 29, 31]. Most of the respondents knew that simple analgesics and combination analgesics may induce MOH. However, almost 30% did not know that triptans may induce MOH and similar to what has been found among primary care physicians in the US and among Norwegian neurology residents, 24% were unaware that opioids may lead to MOH [3, 17].

GP specialists have a more consistent use and knowledge of headache treatment compared to non-specialists, probably due to accumulated clinical experience. In line with previous European epidemiological studies simple analgesics were believed to be the most commonly used medication by chronic headache patients [5, 32, 33]. Sixty percent found MOH to be a clinical challenge among their headache patients. Evidence-based treatment with Brief Intervention for MOH in primary care does exist and it is encouraging that almost all the GPs approached patients with MOH with withdrawal and according to the newly published European Academy of Neurology guidelines [34–36]. However, based on the modest knowledge about which medications may induce MOH, it may be that many headache patients with MOH are still not diagnosed and treated adequately, and may remain unrecognized in primary care. MOH is in principle preventable, thus, identification and information to patients at risk are important [34].

The GPs found “No good treatment options for many patients” to be the main barrier to more optimised headache care. This may be true for certain headache patients, such as patients with frequent or chronic TTH, but at least for migraine, evidence-based treatment options are easily available for most patients in primary care. Headache disorders may be chronic conditions, and patients typically have a need for testing different treatments and long-term follow-up. This may be part of the reason why many GPs find headache patients difficult and demanding, and that they find that the lack of time in general practice contribute to reduced care. Though only 9% mentioned insufficient knowledge as a main barrier, it is obvious, based on the suboptimal management of CTTH and MOH in the present study that more knowledge on these entities is needed.

The societal costs of headache are high, increase with severity of symptoms and referral to specialized care [10, 37]. Thus, improved management in primary health care would be of benefit for both patients and society. As there is still a large knowledge gap about how patients are diagnosed and treated in primary care, further studies among GPs may give supplemental information needed to lay the ground for educational efforts such as systematic headache training in CME groups [16, 21, 38]. Such surveys should be repeated to evaluate time trends and whether specific interventions affect the management and knowledge of headache. In addition, quality indicators of adequate management should be developed and validated specifically for headache in primary care [39]. This may be a first step towards a high-quality, predictable management of headache disorders.

## Conclusion

Most of the GPs follow the national recommendations for migraine, but the clinical knowledge of CTTH and MOH treatment varies. GPs often used MRI in the diagnostic work-up contrary to the recommendations. A more structured headache education for GPs could have direct relevance for better clinical outcomes and reduced costs.

## Supplementary Information

**Additional file 1**

## Data Availability

The authors declare that the data supporting the findings of this study are available within the article.

## Abbreviations

GP: General practitioner
CME: Continuous medical education
ICHD-3: International Classification of Headache Disorders 3rd edition
CTTH: Chronic tension-type headache
MOH: Medication-overuse headache
TACs: Trigeminal autonomic cephalalgias
NSAIDs: Nonsteroidal anti-inflammatory drugs
CGRP: Calcitonin gene-related peptide
GPs: General practitioners
SD: Standard deviations
CI: Confidence interval
IQR: Interquartile range
