# Supplementary material for: The management and clinical knowledge of headache disorders among general practitioners in Norway: a questionnaire survey
Source: J Headache Pain. 2021 Nov 11;22(1):136. doi: 10.1186/s10194-021-01350-3 (PMC8582095; doi:10.1186/s10194-021-01350-3)
Supplement: Supplementary file 1 — Additional file 1 [file 10194_2021_1350_MOESM1_ESM.docx]

***Background questions:***

**Questionnaire on the management of patients with headaches**

1) Age: _______

2) Sex: Man Woman

3) How many years have you worked clinically as a physician (including internship)? ________

4) How many years have you worked as a general practitioner? ________

5) Are you certified as a specialist in general practice? Yes No From which year? _____

6) How many people are on your patient list? _____

7) Approximately how many patient consultations (coded 2ad) do you have in an average day? ________

8) Have you previously participated in continuing education course on headaches? Yes No

***Questions about your own experiences with the treatment of headache patients:***

9) Do you find headaches to be a clinically difficult professional challenge? (rate from 1-4, where 1 is difficult and 4 is simple)? __

10) What type of medicine do you think your chronic headache patients most commonly use to treat their headaches (regardless of headache diagnosis)? (circle one option)

i) Non-prescription medication ii) Prescription medication (C medications) iii) Prescription medication (A/B medications)

| 11) Do you usually use CT/MRI to investigate the following conditions? (check boxes) | Yes | No |
| --- | --- | --- |
| All new-onset headache (not acute) |  |  |
| Headache that does not respond to treatment |  |  |
| Headaches with focal neurological symptoms |  |  |
| Worsening of pre-existing headache |  |  |
| Neck pain with concomitant headache |  |  |
| Patient concerns/anxiety about tumor cerebri/intracranial pathology |  |  |

| 12) (Check one box for each question) | Always | 2 out of 3 times | Half the time | 1 out of 3 times | Never |
| --- | --- | --- | --- | --- | --- |
| a) How often do you use a headache diary when diagnosing a headache? |  |  |  |  |  |
| b) How often do you use a headache diary when following up on headache treatment? |  |  |  |  |  |
| c) If you instruct patients to use a headache diary, how often do you get completed diaries back? |  |  |  |  |  |
| d) How often do you use CT in investigations of new-onset headaches? |  |  |  |  |  |
| e) How often do you use CT in investigations of long-lasting headaches? |  |  |  |  |  |
| f) How often do you find CT to be useful in the investigation of headaches? |  |  |  |  |  |
| g) How often do you use MRI in investigations of new-onset headaches? |  |  |  |  |  |
| h) How often do you use MRI in investigations of long-lasting headaches? |  |  |  |  |  |
| i) How often do you find MRI to be useful in investigations of headaches? |  |  |  |  |  |
| j) How often do you use CT/MRI to alleviate a patient’s concerns/anxiety about tumor cerebri or other intracranial pathology? |  |  |  |  |  |

13) Do you routinely ask your headache patients if they have a high amount of sick leave, reduced social functioning, and disability/reduced quality of life due to headache? (circle one option)

Always 2 out of 3 times Half of the time 1 out of 3 times Never

14) How many of your chronic headache patients (headache >15 days/month) would you estimate overuse acute headache medication? (circle one option)

0-20 % 20-40% 40-60% 60-80% 80-100%

15) How do you typically treat a patient with chronic tension headache (>15 days of tension headache per month)?

- Acute medication? Yes/No If so, preferred medication(s)? ______________________________

- Preventative medication? Yes/No If so, preferred medication(s)? ______________________________

- Physical treatment? Yes/No If so, please specify the preferred treatment(s)? ___________________-

16) How do you typically treat a patient who experiences 1-2 migraine attacks/week?

- Acute medication? Yes/No If so, preferred medication(s)? ______________________________

- Preventative medication? Yes/No If so, preferred medication(s)? ______________________________

- Physical treatment? Yes/No If so, please specify the preferred treatment(s)? ___________________-

17) Which of these medications used in headache treatment would you expect could cause drug overuse headaches/drug induced headaches (check boxes)

|  | Yes | No |
| --- | --- | --- |
| Antiepileptics (valproate, topiramate, gabapentin, lamotrigine, carbamazepine)? |  |  |
| Ergotamines? |  |  |
| Antihypertensives (beta blockers, angiotensin II blockers, calcium blockers, etc.)? |  |  |
| Simple analgesics (paracetamol, ibuprofen, diclofenac, naproxen, etc.)? |  |  |
| Combination drugs (paracetamol/codeine, phenazone/caffeine, paracetamol/caffeine, etc.)? |  |  |
| Opioids? |  |  |
| Antidepressants (tricyclic antidepressants, SSRIs)? |  |  |
| Triptans? |  |  |
| Botox (botulinum toxin A)? |  |  |

18) a) What treatment do you usually suggest for people with medication-overuse headaches? (check one box)

| Nothing in particular |  |
| --- | --- |
| Initiation of preventive medicine without discontinuation of the overused medication |  |
| Discontinuation of the overused medication and initiation of preventive medicine at the same time |  |
| Discontinuation of the overused medication and possible initiation of preventive medicine upon later assessment |  |

| b) Do you usually recommend the following when treating medication-overuse headaches? (check boxes) | Yes | No |
| --- | --- | --- |
| Treatment with acute painkillers/rescue medication |  |  |
| In-patient treatment |  |  |
| Sick leave |  |  |

| 19) Do you experience (check boxes) | Yes | No |
| --- | --- | --- |
| - medication-overuse headache to be a challenge among your headache patients? |  |  |
| - that medication-overuse headache improve in most patients who discontinue the overused medication? |  |  |

20) Which of these conditions (or suspected conditions) do you most often refer to a neurologist (total number of patients/year)? (rank from 1-8, where 1 is most frequently referred)

| Cerebrovascular conditions |  | Multiple sclerosis |  |
| --- | --- | --- | --- |
| Dementia |  | Parkinson’s disease |  |
| Epilepsy |  | Back/neck problems |  |
| Headache |  | Fatigue/ME |  |

21) What do you think of the waiting time for headache patients at the local neurological outpatient clinic? (circle one)

Short Acceptable Long Unacceptable, must refer to private practice specialists

22) How useful is it to refer headache patients to your local neurological out-patient clinic or neurologist? (rank from 1-5, where 1 is very useful and 5 is useless ___

23) What are the most common reasons you refer headache patients to a neurologist? (rank 1-4, where 1 is the most common)

| Diagnostic uncertainty |  |
| --- | --- |
| Suspicion of serious underlying causes |  |
| Treatment failure/lack of good treatment options in general practice |  |
| The patient's own wishes/expectations |  |
| Any other reasons, please specify: |  |

24) How useful (diagnosis, information and advice) are notes from a neurologist in a patient’s records when you are following up with headache patients? (rank from 1-5, where 1 is very useful and 5 is useless) ___

25) How do you perceive the collaboration with neurologists regarding your headache patients? (circle one option)

Good Mostly good OK Bad Nonexistent

26) What do you believe would contribute to better collaboration with your local neurological outpatient clinic or neurologist when treating headache patients (rank from 1-4, where 1 is the most important measure and 4 the least important)?

| Telephone time for the GPs to have direct access with neurologists |  |
| --- | --- |
| Direct electronic communication with neurologists |  |
| Guidelines with recommendations for referrals |  |
| Visiting program for GPs at the Department of Neurology |  |
| Other, please specify: |  |

27) What do you believe are the main barriers to optimised treatment and management of your headache patients? (rank from 1-6, with 1 as the most important barrier obstacle and 6 as the least important)

| Your own insufficient knowledge of headache medicine |  |
| --- | --- |
| Headache patients are difficult and demanding |  |
| Too little time in general practice |  |
| There are no good treatment options for many patients |  |
| Lack of financial incentive to manage these patients in general practice |  |
| Too little support from, or collaboration with, specialists |  |

28) How do you rate your own knowledge of these headache disorders (check boxes):

|  | Good | Average | Minimal |
| --- | --- | --- | --- |
| - Migraine |  |  |  |
| - Tension headache |  |  |  |
| - Cluster headache / trigeminal autonomic cephalalgias |  |  |  |
| - Medication-overuse headache |  |  |  |

| 29) When you have headache patients, how often do you use (check boxes) | Always | 2 out of 3 times | Half the time | 1 out of 3 times | Never |
| --- | --- | --- | --- | --- | --- |
| a) National recommendations for the treatment of headaches (NEL) |  |  |  |  |  |
| b) BMJ Best Practice/Up to date via the Norwegian Electronic Health Library |  |  |  |  |  |
| c) The International Classification of Headaches Disorders (ICHD) |  |  |  |  |  |
| d) Google searches |  |  |  |  |  |

30) As part of a new specialist education programme, a new course structure is being developed for continuing medical education

a) Which type of course do you find has the best learning outcomes for you (rank from 1-4, where 1 represents the best learning outcome and 4 the worst)

Full-day course (1-5 days)__ Evening course over several weeks___ Course in a small group___ Online course___

b) What kind of lecturer do you prefer to have in courses in general medicine (rank from 1-3, where 1 is the best and 3 the worst)

Hospital specialist___ General practitioner with specialist knowledge in the field ___ Joint lectures (hospital doctor and general practitioner) ___

c) What are four topics you would like to see on the programme of a clinical course on headaches in general medicine?

d) What are four neurology topics you would like to see on the programme of a clinical course on neurology in general medicine?
